# Successful treatment with yttrium-90 microspheres in a metastatic breast cancer patient and sclerosing cholangitis

**DOI:** 10.2144/fsoa-2021-0015

**Published:** 2021-06-04

**Authors:** Aurélie Louvet, Cédric van Marcke, Philippe D'Abadie, Emmanuel Seront

**Affiliations:** 1 Department of Medical Oncology, Cliniques Universitaires Saint-Luc, Brussels, Belgium; 2 Department of Nuclear Medicine, Cliniques Universitaires Saint-Luc, Brussels, Belgium; 3 Department of Medical Oncology, Hopital de Jolimont, Haine Saint Paul, Belgium

**Keywords:** breast cancer, liver metastases, metastasis-directed therapies, trans-arterial radioembolization, yttrium-90

## Abstract

Breast cancer is the most common malignancy occurring in women worldwide. More than 90% of patients present with localized disease are treated with curative intent; however, recurrence can occur with development of metastatic lesions. Frequently associated with extra-hepatic lesions, localized treatments (surgery or stereotaxic body radiotherapy) are rarely proposed in liver lesions. ^90^Y radioembolization has extensively been evaluated in colorectal cancer, but its role in breast cancer with isolated liver metastases remains largely unknown. Pre-existing liver diseases are known risk factors for ^90^Y induced liver toxicity. Not considered as an excluding factor for this treatment, data are limited regarding its safe use with cholangitis. We report a successful control of liver metastases by ^90^Y radioembolization in a breast cancer patient.

## Lay abstract

Breast cancer is the most common malignancy occurring in women worldwide. Metastasis-directed therapies are more and more often offered to patients in order to delay systemic treatments; however, their role in improving survival remains unknown. Trans-arterial radioembolization with ^90^Y appears to be an interesting strategy in breast cancer with isolated liver metastases, even if its survival benefit remains largely unknown. Safety of trans-arterial radioembolization also remains under investigated in patients with previous liver disease. We report a successful control of liver metastases by ^90^Y radioembolization in a breast cancer patient.

Breast cancer is the most frequent cancer and one of the leading causes of death in women worldwide [1]. Although the majority of breast tumors are treated with a curative intent, cancer resurgence can occur with development of metastases. Liver metastases are detected in 15% of metastatic breast cancer patients but these metastases are in the majority of cases associated with other extra-hepatic localizations, including lymph nodes, bone or lung [2].

Systemic treatments, including endocrine therapy and chemotherapy, remain the cornerstone of patients with metastatic breast cancer. Many improvements have been made in this setting and addition of cyclin dependent kinase (CDK) 4/6 inhibitors or a PI3K inhibitor improves prognosis of patients with hormone positive tumors compared with endocrine therapy alone. However, resistance develops systematically, requiring subsequent therapies with limited response and cumulative toxicities [3]. There is more and more evidence that isolated metastases could in some cases represent a window of opportunity for localized treatment such as stereotaxic ablative body radiotherapy, thermal ablation or surgery [3–6]. These strategies, however, have not been shown to provide a significant survival advantage and require a correct selection of patients. Only 5% of metastatic breast cancer patients present with isolated liver metastases, not associated with extra-hepatic metastases. It remains unknown if in these patients metastasis-directed treatment could improve prognosis and/or delay initiation of systemic treatment.

Trans-arterial radioembolization (TARE) with ^90^Y, by exploiting the hepatic arterial flow, appears as an interesting way to target liver metastases. Radioactive microspheres are injected in the hepatic artery and deposited through the arterial network in the most vascularized liver areas, locally releasing ionizing radiations. TARE appears thus as a promising liver-directed therapy in patients with hepatic tumors. TARE is currently approved, when surgery is not feasible, in the treatment of primary hepatocellular carcinoma and liver metastases from colorectal cancer [7]. TARE is often well tolerated with a low risk to develop a post-therapy liver failure termed radioembolization-induced liver disease (REILD) [8]. The role of TARE in liver metastases from breast cancer remains to be determined, as only small patient cohorts have been reported [9–14]. Even if feasibility and safety have been demonstrated, many issues involve the TARE feasibility in patient with concurrent liver disease such as cirrhosis or sclerosing cholangitis.

Here, we report the safe and successful use of ^90^Y radioembolization in a patient with isolated breast cancer liver metastases and a history of sclerosing cholangitis.

## Case presentation

A 64-year old woman was diagnosed in 2001 with a localized right breast cancer (estrogen receptor Allred score 8/8, progesterone receptor Allred score 0/8, absence of HER2 expression as assessed by immunohistochemistry, Ki67 60%) and was treated with surgery, chemotherapy (six courses of anthracycline-based chemotherapy), radiotherapy and endocrine therapy (tamoxifen) for 5 years. Her past medical history included a primary auto-immune cholangitis that was diagnosed in 2008 based on grade 2 alanine aminotransaminase and aspartate aminotransferase liver and cholestatic tests (alcaline phosphatase) and gamma-glutamyl transpeptidase disorders, positive antinuclear antibodies (1/320) and cholangiography; there was no sign of cirrhosis at fibroscan. Ursodeoxycholic acid therapy resulted at this time in normalization of cholestatic and liver tests and this benefit is still maintained. In 2014, she described right brachialgia with decreased arm sensitivity. Thoraco-abdominal computed tomography (CT) and cervical MRI showed a right axillary lymph node involving the brachial plexus. Biopsy confirmed resurgence of breast cancer with similar histological characteristics than previously reported. ^18^Fluoro-deoxy-glucose (FDG)-PET-CT did not show any other distant lesion. Six cycles of chemotherapy (paclitaxel and gemcitabine) led to a partial response, allowing a complete surgical resection of the lymph node. Letrozole was then started as ‘adjuvant’ treatment.

In August 2019, four liver lesions were detected on thoraco-abdominal CT. Biopsy confirmed the resurgence of breast cancer (estrogen receptor 8/8, progesterone receptor 0/8, no HER-2 expression, KI67 60%). Surgery was deemed unfeasible due to localization of the lesions. A CDK4/6-inhibitor (palbociclib) was associated with fulvestrant, resulting in minor regression of the lesions (<30% of size decrease based on RECIST criteria). In May 2020, thoraco-abdominal CT showed apparition of multiple bilobar liver lesions (around ten), without any other extra-hepatic lesion. Laboratory tests were within normal range. As metastases were restricted to the liver and in order to delay subsequent line of systemic treatment, TARE was performed in June 2020 using ^90^Y loaded resin microspheres (SIR-Spheres^®^, Sirtex Medical Ltd, Australia). Baseline liver tests such as aspartate aminotransferase, alanine aminotransaminase, gamma-glutamyl transpeptidase, alkaline phosphatase (ALP) and bilirubin were within normal range. The treatment was well tolerated by the patient, without any adverse event. The planned radioactivity was determined using the partition model with a dose of 50 Gy to the nontumoral liver. The treatment was performed by injection in the right liver artery of 0.67 and 0.38 GBq in the left liver artery.

**Figure 1.**
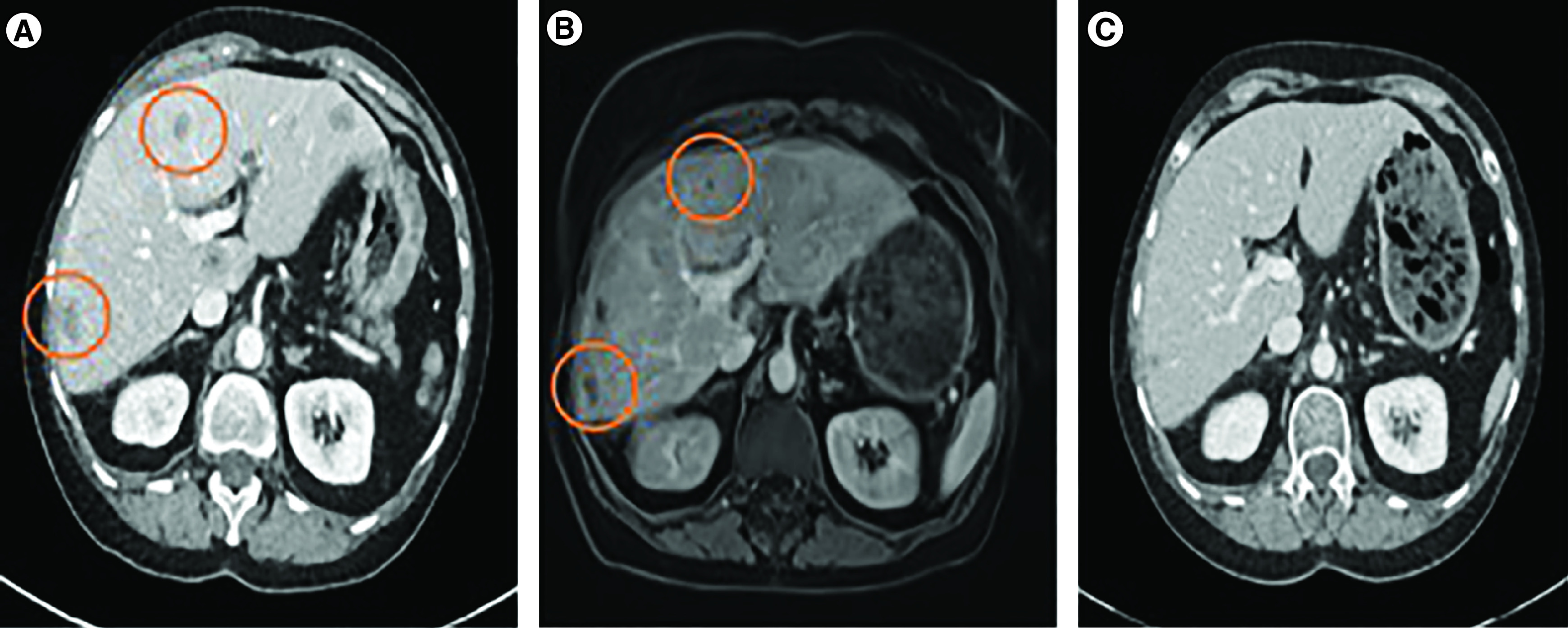
Evolution of liver metastatic lesions on baseline and after trans-arterial radioembolization. **(A)** Abdominal CT before TARE. **(B)** Abdominal MRI performed 2 months after TARE, showing decrease of all previously described lesions. ^18^FDG-PET did not show any metabolic lesion. **(C)** Abdominal CT performed 6 months after TARE, showing disappearance of previously described lesions. ^18^FDG-PET did not show any metabolic lesion. CT: Computed tomography; FDG: Fluoro-deoxy-glucose; TARE: Trans-arterial radioembolization.

Four weeks after TARE, thoraco-abdominal CT showed stability of known hepatic metastases and absence of new extra-hepatic lesions; laboratory test was normal, including hemogram and liver tests. In order to maintain antitumoral pressure, alpelisib was added to fulvestrant, as a *PIK3CA* hotspot mutation was detected by next-generation sequencing (NGS) test of the liver biopsy of August 2019. However, 9 days after alpelisib initiation, our patient presented with epistaxis and grade 4 common terminology classification for adverse events thrombopenia, requiring immediate interruption. Thrombopenia corrected spontaneously in 2 weeks and fulvestrant was maintained as single agent. Two months after TARE, MRI showed decrease in size and number of hepatic lesions (80% decrease in size of largest lesions based on RECIST criteria) and ^18^FDG-PET-CT showed absence of metabolic activity (Figure 1). In January 2021, we observed a complete radiological and ongoing metabolic response on thoraco-adominal CT and ^18^FDG-PET-CT (Figure 1). The patient is currently still on fulvestrant alone (progression-free survival of 10 months, ongoing).

## Discussion

Metastasis-directed treatment such as surgery, stereotaxic ablative body radiotherapy or thermal ablation have been shown to improve recurrence free survival without any significant survival advantage; however, these strategies should be considered in highly selected patients with the goal of providing time off systemic chemotherapy. Important issues involve the difficulties to perform complete resection and the volume of the metastases, particularly in liver [4–6].

TARE is commonly used for the treatment of metastatic colorectal cancer and unresectable hepatocellular carcinoma. However, its role in breast cancer liver metastases is not clearly determined, probably due to the fact that, in breast cancer, liver metastases are often associated with a more extensive disease spread making local strategies less appropriate. However, different case series have reported potential antitumoral efficacy with a disease control rate (complete response, partial response and stable disease) reaching 77%, including 7% of complete response. Overall survival ranged from 3.6 to 20.9 months with an estimated mean survival of 11.3 months [9–14]; however, these results should be confirmed in randomized controlled trials.

Our case is interesting as it highlights the efficacy of TARE in controlling liver involvement of breast cancer, even after progression on CDK4/6 inhibition and fulvestrant. TARE with ^90^Y resulted in our patient in a complete radiologic response at the 2–6 month CT scan, without metabolic activity at concomitant ^18^FDG-PET-CT, reflecting a rapid and sustained activity of microspheres. At 10 months, the patient is still free of progression, with complete metabolic response on ^18^FDG-PET-CT.

In order to control potential extra-hepatic spreading, alpelisib was added to fulvestrant 1 month after TARE. However, rapid onset high-grade hematological toxicity led to its interruption, suggesting that alpelisib did not play a role in the significant radiological response observed at 2–6 months.

Whether alpelisib–fulvestrant without TARE could have resulted in similar liver response remains unknown; however, in our case, TARE presented some advantages. First, it was very well tolerated, in contrast to alpelisib that led to life-threatening toxicity. Second, TARE improved local control for at least 10 months (still ongoing), which is highly significant in heavily pretreated patients. Third, due to this sustained local control, TARE is allowing the delay of subsequent treatment initiation such as chemotherapy or endocrine-targeted agents association that could in some patients deteriorate the quality of life.

Our patient has a history of auto-immune sclerosing cholangitis that was well controlled with ursodeoxycholic acid since 2008. Liver biology was frequently controlled after TARE, as secondary liver toxicity can occur. This adverse event, termed REILD, usually consists of icterus and ascites appearing 1–2 months after treatment in the absence of tumor progression or bile duct occlusion. REILD is more likely to occur in patients with history of cirrhosis or after the exposure to anticancer chemotherapy before TARE [8]. Our patient had no cirrhosis and no recent exposure to cytotoxic agents except CDK4/6 inhibitors. No liver perturbation was observed in our patient after ^90^Y loaded microspheres administration, despite the pre-existing immune cholangitis. Moreover, this suggests that TARE could be feasible after exposure to CDK4/6 inhibitors and in patients with sclerosing cholangitis and normal liver tests. However, many questions remain concerning patients with active sclerosing cholangitis and abnormal liver tests, which was not the case for this patient. The optimal imaging assays for response evaluation remains also unknown, but it was shown that early response to ^18^F-FDG-PET based on standardized uptake level maximal (SUVmax) could significantly predict survival in this patient population [15,16].

## Conclusion

^90^Y treatment can be considered as part of a management strategy for isolated breast cancer liver metastases, even in patients with pre-existing cholangitis and after exposure to CDK4/6 inhibitors.

## Future perspective

Metastasis-directed therapies appear as a promising strategy in improving outcome of patients with metastatic breast cancer. Even if the general survival advantage is not proven, local recurrence free survival is improved. However, this requires an ideal selection of patients with real localized metastatic disease. There is thus an urgent medical need to develop ways to evaluate whether there is a need for a systemic treatment or whether there is a window of opportunity to treat locally the metastases.

**Executive summary**

- Trans-arterial radioembolization (TARE) with ^90^Y resulted in our patient in a complete radiologic response of liver metastases and result in a 10-month recurrence free survival (still ongoing).
- TARE with ^90^Y was well tolerated in this patient with previous history of sclerosing cholangitis.
- TARE with ^90^Y allowed us to delay in our patient a highly systemic treatment that was shown to be temporary life threatening.
- TARE with ^90^Y appears thus as a promising strategy to consider in some selected patients with localized liver metastatic disease.
